# The Callus of *Phaseolus coccineus* and *Glycine max* Biotransform Flavanones into the Corresponding Flavones

**DOI:** 10.3390/molecules25235767

**Published:** 2020-12-07

**Authors:** Monika Dymarska, Tomasz Janeczko, Edyta Kostrzewa-Susłow

**Affiliations:** Department of Chemistry, Wrocław University of Environmental and Life Sciences, Norwida 25, 50-375 Wrocław, Poland; janeczko13@interia.pl (T.J.); edyta.kostrzewa-suslow@upwr.edu.pl (E.K.-S.)

**Keywords:** callus cultures, biotransformation, flavanone, flavone, 5-methoxyflavanone, 5-methoxyflavone, 6-methoxyflavanone, 6-methoxyflavone

## Abstract

In vitro plant cultures are gaining in industrial importance, especially as biocatalysts and as sources of secondary metabolites used in pharmacy. The idea that guided us in our research was to evaluate the biocatalytic potential of newly obtained callus tissue towards flavonoid compounds. In this publication, we describe new ways of using callus cultures in the biotransformations. In the first method, the callus cultures grown on a solid medium are transferred to the water, the reaction medium into which the substrate is introduced. In the second method, biotransformation is carried out on a solid medium by growing callus cultures. In the course of the research, we have shown that the callus obtained from *Phaseolus coccineus* and *Glycine max* is capable of converting flavanone, 5-methoxyflavanone and 6-methoxyflavanone into the corresponding flavones.

## 1. Introduction

Flavonoids are a numerous and diverse group of secondary plant metabolites. Research on cell lines, animals and humans prove that flavonoids have a strong and diverse biological potential, which allows the search for molecules that could be used in medicine and pharmacy [1,2].

Flavones are one of the largest groups of flavonoid compounds. They show many attractive biological activities: anti-inflammatory, antiallergic, antiviral, anticancer, hepatoprotective, and cardioprotective [3]. The presence of the double bond between the C2-C3 carbon atoms that distinguishes flavones from their structural analogues, flavanones, is often crucial for their properties. Higher biological potential of flavones has been proven, inter alia, in studies of antioxidant activities [2,4,5,6]. Flavones are usually also more active in tests for antifungal [7] and antiparasitic properties [8].

The possibilities of obtaining secondary plant metabolites are limited mainly by the availability of natural sources. The use of plant cultures for the production of secondary metabolites has many advantages: compounds can be extracted and purified easier, it is possible to obtain new products that do not occur in nature, and production becomes independent of external factors, such as weather and changing seasons [9,10,11].

In vitro plant cultures can potentially be used as biocatalysts in the biotransformations of structurally diverse substrates. Plant enzyme systems are able to carry out hydroxylation, oxidation of hydroxyl groups, reduction of carbonyl groups, reduction of the double bond, and hydrolysis or introduction of a sugar unit [12,13,14,15,16]. In the so far described examples of flavonoid biotransformations, glycosylation is the prevailing type of reactions.

Currently, hundreds of enzymes have industrial applications in biocatalysis and biotransformation processes. The source of more than half of those enzymes is fungal strains, more than a third are derived from bacteria, while the others come from animals and plants [17].

There are many reports in the literature on microbial transformations of flavonoid compounds. For several years, our team has been utilizing entomopathogenic filamentous fungi as biocatalysts [18,19,20,21,22,23]. Using these microorganisms, we mainly observe the attachment of the 4-*O*-methylglucopyranose molecule to the flavonoid aglycon.

Marine microorganisms are an interesting tool for the biotransformation of flavonoid compounds or a source of enzymes that can be used in modifying the structures of chemical compounds [24]. These organisms show the ability to live in an environment with variable temperature, extreme pH values, high salt concentration, and high pressure. For this reason, they are characterized by the production of a variety of unique enzymes with a wide range of potential industrial applications [17]. Marine microorganisms are able to carry out various modifications of flavonoid compounds, e.g., hydroxylation, hydrolysis of glycosidic bonds, or reduction of chalcones [17,25]. The last reaction is of particular interest because it leads to highly bioactive dihydrochalcones with possible use as sweeteners in the food industry [17,26,27].

A strongly developed trend in research on the production of flavonoid compounds is the use of genetically modified microorganisms, both prokaryotic (*Escherichia coli*) and eukaryotic (*Saccharomyces cerevisiae*, *Yarrowia lipolytica*). In this way, it is possible to obtain flavonoids such as stilbene, naringenin, eriodictyol, and taxifolin [28,29].

In their extensive review, Wang et al. present the versatility of the enzymatic systems of microorganisms in the biotransformation of flavonoids, describing more than 200 flavonoid compounds [30]. An example of a flavonoid biosynthetic pathway frequently used in the literature is that leading to the formation of naringenin. In plants, this flavanone is converted to flavone by flavone synthase I (FNS I) or flavone synthase II (FNS II). Flavanone 2-hydroxylase (F2H) catalyzes the hydroxylation of flavanones to 2-hydroxyflavanones, which are subsequently converted to flavones [31]. Plant cultures have a tempting potential for industrial use. Despite this, they are still used less frequently than, for example, cultures of microorganisms. Significant obstacles are problems with the multiplication of a sufficient amount of tissue in the suspension cultures and the high cost of their production [32,33,34]. The current publication presents a method for the efficient acquisition of large amounts of plant biomass. It can potentially be used in biotransformation processes carried out in water, which simplifies the procedure and reduces its cost. Our research also proves that, for some callus cultures, it is possible to carry out biotransformation on a solid medium, which is even more cost-effective. The idea that guided us in our research was to evaluate the biocatalytic potential of callus tissue towards flavonoid compounds. The callus cultures of *Phaseolus coccineus* L. (Leguminosae) and *Glycine max* (L.) Merrill (Leguminosae) demonstrate the ability to biotransform flavanones (flavanone, 5-methoxyflavanone, and 6-methoxyflavanone) into potentially more biologically active flavones. The process yields were not satisfactory. However, the aim of our work was to show new catalytic abilities of callus cultures of *P. coccineus* and *G. max*. Further research is needed to determine the application nature of the obtained plant cultures.

## 2. Results and Discussion

To obtain callus cultures of *P. coccineus* and *G. max*, the seeds of these plants were sterilized and transferred to sterile medium according to the procedure described in Section 3.2.1. (seed sterilization). The germinated plants were then aseptically cut and placed on sterile media according to the method described in Section 3.2.3. (seed germination and callus induction).

**Figure 1:**
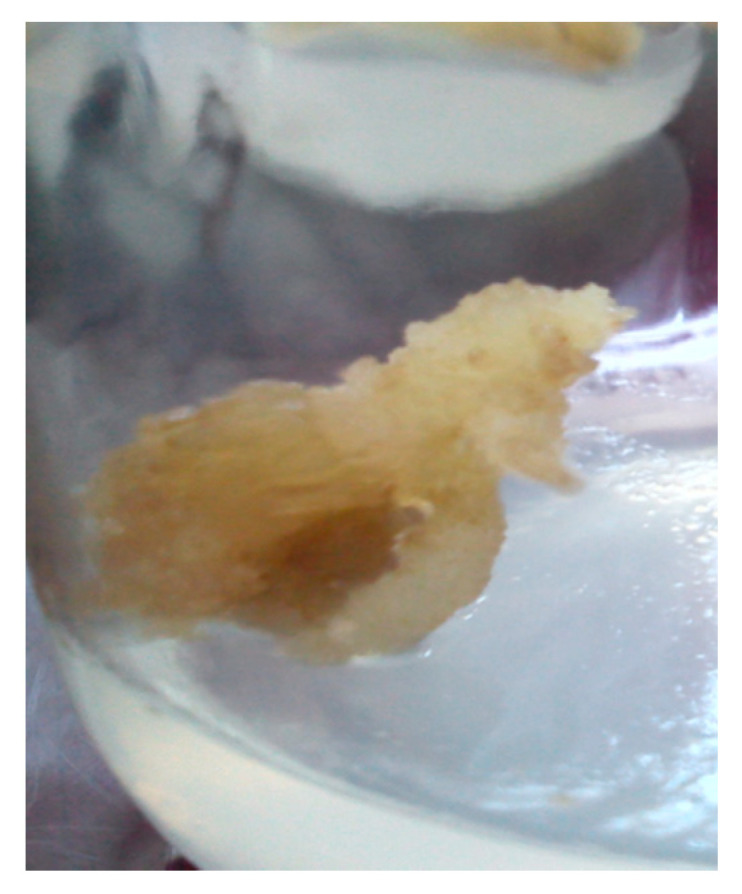
*G. max* callus forming on an explant.

**Figure 2:**
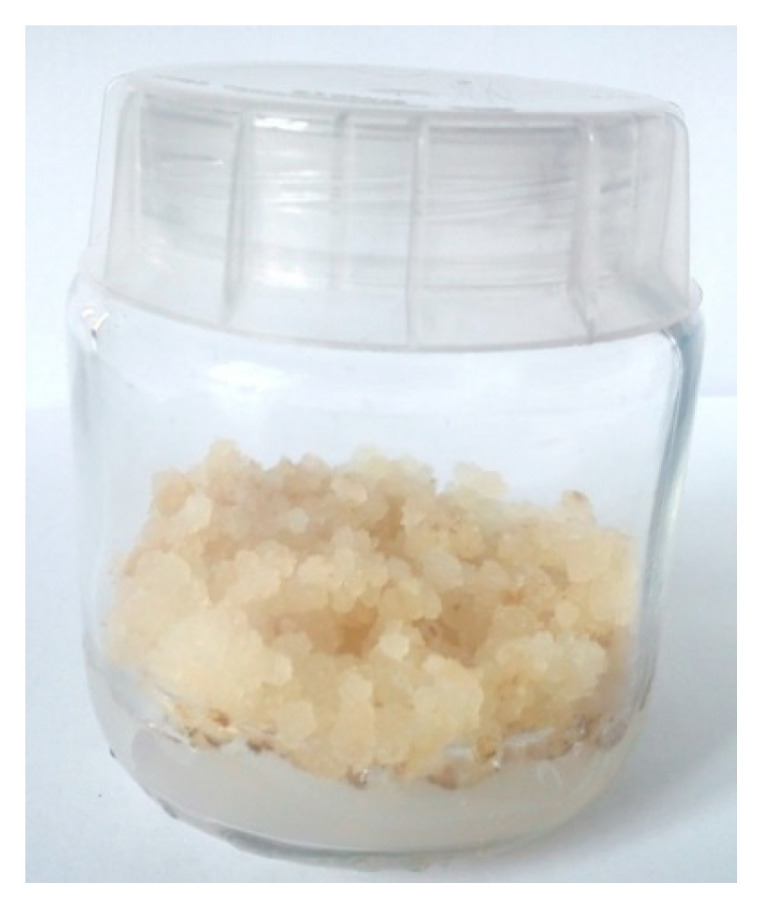
*P. coccineus* 10-day callus culture.

After approximately one week, significant amorphous tissue formation was observed at the ends of the explants in contact with the medium (Figure 1). The tissue was passaged onto fresh medium containing growth regulators. The obtained callus of *P. coccineus* and *G. max* was homogeneous and loose, characterized by a cream colour and intensive growth (Figure 2). In the following passages, approximately 6–8 g of callus tissue was formed per vessel. The calluses of both plants were used for experiments in the aquatic environment. Only *P. coccineus* cultures developed on a solid substrate with flavonoid compounds deposited on the surface. Under these conditions, the *G. max* callus did not grow. Rather, it quickly darkened and died out.

After seven and 14 days, the pH of the aqueous plant cultures was measured. The cultures to which the flavonoid compound was added had a pH in the range of 4.9–5.1. The callus suspended in water without flavonoids showed a pH above 6. After 14 days, the aqueous callus cultures began to darken and die; their pH increased to about 8. However, cultures with the added flavonoids remained fair, and their pH did not increase. On this basis, it can be concluded that the addition of flavonoid compounds to aqueous plant cultures prolongs their stability, probably due to the antioxidant activity of flavonoids.

**Scheme 1:**
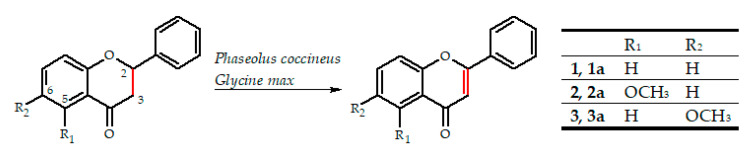
Biotransformations of flavanones in *P. coccineus* and *G. max* callus cultures.

All used flavanones were biotransformed into the corresponding flavones (Scheme 1).

The biotransformation efficiencies in *P. coccineus* cultures were low, not exceeding 2.3% (Table 1). However, in the case of *G. max* and 6-methoxyflavanone as a substrate on the 14th day of biotransformation, the yield was 7.5% (Table 2).

In order to exclude the spontaneous conversion of flavanones to flavones, the stability of all substrates was checked in water and also in a buffer with a pH equal to 5. In no case was a trace of flavones detected.

When 5-methoxyflavanone was used as a substrate, in an experiment using the growing *P. coccineus* callus on a solid MS medium as a biocatalyst, a higher yield was obtained than in aqueous cultures (1.3% vs. 0.2%) (Table 3). The callus was separated from the MS medium and analyzed separately. The presence of flavones was demonstrated only in the plant mass, which proves the effective uptake of flavonoid compounds from the medium and biotransformation in the biocatalyst cells.

Naringenin as a natural flavone precursor is most often used in studies of the formation of the C2-C3 double bond. Cell-free extracts from primary leaves of *Petroselinum hortense* Hoffm. (parsley) were used to oxidation naringenin to apigenin [35]. Later, this plant served as a source of flavone synthase type I (FNS-I), which was expressed in *Arabidopsis thaliana* lacking the flavone biosynthetic pathway. Studies have resulted in the efficient conversion of naringenin to apigenin [36].

The complex of enzymes responsible for the synthesis of flavones from simple precursors has been identified, isolated and expressed in *Saccharomyces cerevisiae* cells. In this way, it was possible to obtain chrysin, apigenin, and luteolin [37]. In another study, eriodictyol, naringenin, and liquiritigenin were biotransformed to the corresponding flavones directly by the recombinant cytochrome P450 proteins from *Lonicera japonica* Thunb. and *L. macranthoides* Hand.-Mazz [38]. Luteolin was obtained from eriodictyol, 7,4′-dihydroxyflavone from liquiritigenin, quercetin from dihydroquercetin, and apigenin from naringenin, when Cytochrome P450 cDNAs, AFNS2 and TFNS5, from snapdragon and torenia petal cDNA were expressed in yeast [39].

We did not find studies describing the use of callus cultures to obtain flavones from the corresponding flavanones. Kostrzewa et al. described the formation of flavone, 6-hydroxyflavone and 7-hydroxyflavone in fungal cultures of *Aspergillus niger*. The *A. niger* 13/5 strain converted flavanone to flavone with 33% efficiency. The same strain allowed the conversion of 6-hydroxyflavanone to 6-hydroxyflavone with a yield of 76%, and 7-hydroxyflavanone to 7-hydroxyflavone with a yield of 98% [40,41,42].

FNS I and FNS II, responsible for converting flavanones to flavones in plants, are characterized by different catalytic mechanisms. The FNS I class belongs to the superfamily of soluble Fe^2+^/2-oxoglutarate-dependent dioxygenases. FNS II enzymes correspond to cytochrome P450 monooxygenases. This broad group of membrane-bound enzymes is widespread among the higher plants. The enzymes in dicotyledonous plants belong to CYP93B subfamily, while in the monocotyledons to the CYP93G subfamily [43].

The desaturation of the flavanones’ C-ring by FNS I enzymes includes two steps: elimination of the C-3 β-configured hydrogen and subsequent elimination of the C-2 hydrogen. Studies of FNS I activity using ^14^C-radiolabeled substrates confirmed that flavanones’ conversion to flavones occurs without detectable reaction intermediates [43]. On the other hand, FNS II, depending on their plant origin, can convert flavanones to flavones through an intermediate product - with a hydroxyl group attached to C2 or without intermediate products. In the latter case, the use of 2-hydroxynaringenin as substrate did not result in the formation of flavones [44]. In our research, we did not observe the formation of potential reaction intermediates. The explanation of the mechanism of the reactions presented in the publication requires further research.

Biotransformation is the body’s way of dealing with potentially toxic xenobiotics. In mammals, hepatic cytochrome P450 enzymes are mainly responsible for detoxification processes [45,46]. In the introduction to the publication, we presented microorganisms as a useful tool in the biotransformation of flavonoid compounds. Many of them carry out microbial transformations with high efficiency. Microorganisms such as the filamentous fungi of the genus *Cunninghamella* can serve as models for the mammalian metabolism of xenobiotics [47], including flavonoids [48]. Primarily due to the similarities in the structure and functioning of the cytochrome P450 enzymes of microorganisms and mammals [49,50,51]. Microorganisms of the genus *Cunninghamella* can be used in the pharmaceutical industry in early ADME-Tox assays [52].

Flavonoid compounds are not xenobiotics for plants. Our hypothesis, explaining the low efficiency of the biotransformations described in this publication, is that the flavonoid compounds are not recognized by callus cells as potentially toxic compounds. Therefore, although they are taken up by the cells, they are not further transformed. It is also possible that the flavonoid compounds used in the experiment acted as inhibitors of cytochrome P450 [46,53,54] and, therefore, have only been converted to a small extent.

The efficiency of biotransformation mediated by the cytochrome P450 subunits can be increased by using cytochrome P450 inducers such as beta-naphthoflavone [24,55,56,57,58].

Stress factors strongly influence the production of secondary metabolites in plants. The use of elicitors such as methyl jasmonate, salicylic acid, and glutathione, chitosan is a known way to increase the production of flavonoid compounds in plant cultures in vitro [59,60,61,62]. Ultraviolet radiation has a similar effect on the accumulation of flavonoid compounds in plants [63,64,65,66,67].

The use of any of the above methods or their combinations could also positively affect the biotransformation process of flavonoid compounds in the callus cultures discussed in this publication. Callus cultures have a high and diverse potential for industrial use [14]. Callus cultures and plant suspension cultures often differ in terms of growth rate and productivity. Plant cells are large and usually grow in clusters. This makes it very difficult to assess the culture density by counting cells under the microscope, as well as turbidimetric or light scattering measurements [34,68]. The use of callus cultures transferred to water enables an easy and more accurate standardization of the biotransformation process because each time a precisely defined mass of the biocatalyst can be used. Another advantage of callus cultures over suspension cultures is their higher stability. They are often more efficient sources of metabolites for pharmaceutical use [69].

In our work, we only describe preliminary studies, but on their basis, it can be concluded that it is purposeful to search for plant biocatalysts among other plant species. By using plant cultures, obtaining new flavonoid derivatives useful in the pharmaceutical, food, or cosmetic industries will be efficient, ecological, and more widely accepted and desired by potential consumers.

## 3. Materials and Methods

### 3.1. Chemicals

The substrates for biotransformations, as well as chemical standards were purchased from Sigma Chemical Company (St. Louis, MO, USA).

1. Flavanone (**1**)
  C_15_H_12_O_2_, *t*_R_ 19.53.
2. 5-Methoxyflavanone (**2**)
  C_16_H_14_O_3_, *t*_R_ 16.72.
3. 6-Methoxyflavanone (**3**)
  C_16_H_14_O_3_, *t*_R_ 20.17.
4. Flavone (**1a**)
  C_15_H_10_O_2_, *t*_R_ 17.55.
5. 5-Methoxyflavone (**2a**)
  C_16_H_12_O_3_, *t*_R_ 15.37.
6. 6-Methoxyflavone (**3a**)
  C_16_H_12_O_3_, *t*_R_ 18.41.

### 3.2. Plant Cultures

#### 3.2.1. Seed Sterilization

The seeds of the runner bean cultivar Piękny Jaś (*P. coccineus*) or soy (*G. max*) were transferred to a beaker with distilled water for 30 min. Subsequent operations were performed under the laminar chamber. The seeds were transferred to a beaker containing 70% ethanol for 30 s to degrease the surface of the seeds before further sterilization. The ethanol was poured off and the seeds were transferred to a conical flask with 1% sodium hypochlorite solution and shaken for 12 min. After this time, the sodium hypochlorite solution was poured off, and the seeds were rinsed with sterile distilled water four times for 5 min.

#### 3.2.2. Seed Ggermination Medium

Briefly, thirty g/L sucrose and 7 g/L agar were added to the standard amount of Murashige and Skoog (MS) medium. The pH was adjusted to 5.8, and the medium was autoclaved at 121 °C for 15 min.

#### 3.2.3. Seed Germination and Callus Induction

Seeds on the MS medium were placed in the dark at 30 °C for five days to germinate. After this time, the newly formed sprouts were cut from the seeds, cut into 3-mm pieces, cut lengthwise, and transferred to the MS medium prepared as above and supplemented before sterilization with 6-benzylaminopurine and 1-naphthaleneacetic acid at concentrations of 1 mg/L. Growth regulators were selected on the basis of the work of Vaquero et al. [70]. The explants were placed in the dark at 30 °C for ten days to form callus tissue.

### 3.3. Biotransformations

#### 3.3.1. Biotransformations in Water

Briefly, fourteen-day-old callus cultures were used in the experiments. Thereby, ten g of callus cells were transferred under sterile conditions into a 100 mL conical flask with 30 mL of sterile distilled water with a pH of 6.6. The substrate (3 mg) dissolved in 0.5 mL of tetrahydrofuran was added to the biocatalyst. The transformation was carried out at 30 °C with continuous shaking. Samples were taken after 7 and 14 days.

#### 3.3.2. Biotransformations on Solid Medium

Biotransformations were performed by the developing callus tissue of *P. coccineus*. Plant tissue culture jars with a volume of 150 mL contained 25 mL of the agar solidified medium described above (containing growth regulators) were used. On the surface of the medium under the laminar chamber, 5 mg of the substrate suspended in tetrahydrofuran was evenly applied. After the solvent had evaporated, a small amount of callus tissue was transferred to the surface of the medium and spread evenly. Cultures were incubated at 30 °C in the dark for 21 days.

#### 3.3.3. Extraction of Biotransformation Products

In the case of biotransformations in water, the conical flask’s entire contents were transferred to a round-bottom flask, then frozen and lyophilized. The metabolites were extracted with 70% methanol solution (sonication for 15 min, shaking for one hour). After filtration, the solvent was removed using a vacuum evaporator, and the course of the biotransformations was analyzed by chromatographic methods.

In the case of biotransformations on solid medium, the callus was analyzed separately. Extraction was performed as described above.

#### 3.3.4. Assessment of Substrate Stability

The stability of the substrate was tested under the conditions in which the biotransformations were carried out (without the presence of the biocatalyst): in water, on a solidified agar medium, and in an acetate buffer at pH 5.

### 3.4. Analysis

The course of the biotransformation was evaluated by chromatographic methods (TLC, HPLC). A preliminary analysis of the course of the biotransformation was carried out using TLC. Planar chromatography was performed on TLC Silica gel 60/Kieselguhr F254 plates (Merck, Germany). The developing system was a mixture of hexane/ethyl acetate (1:1 *v/v*). Compounds were visualized using 5% AlCl_3_ solution in ethanol. The plates were observed at two wavelengths: 254 and 365 nm.

The exact course of the biotransformation process was determined on the basis of high performance liquid chromatography (HPLC). HPLC analyses were performed using a Waters 2690 instrument equipped with a Waters 996 photodiode array detector, using an ODS 2 column (4.6 × 250 mm, Waters) and a Guard-Pak Inserts µBondapak C18 pre-column. Separation conditions were as follows: gradient elution, using 80% of acetonitrile in 4.5% formic acid solution (eluent A) and 4.5% formic acid (eluent B); flow, 1 mL/min; detection wavelength 280 nm; program: 0–7 min, 10% A 90% B; 7–10 min, 50% A 50% B; 10–13 min, 60% A 40% B; 13–15 min, 70% A 30% B; 15–20 min 80% A 20% B; 20–30 min 90% A 10% B; 30–40 min, 100% A.

The biotransformation products were identified by comparing their retention times and UV maxima with the corresponding standards (supplementary materials).

Quantitative analysis was performed with standard curves using HPLC.

## 4. Conclusions

The method of producing callus cultures of *P. coccineus* and *G. max*, described in the publication, makes it possible to obtain potential biocatalysts for biotransformation processes, as well as a source of bioactive compounds. The described callus cultures are capable of biotransforming flavanones and converting them into the corresponding flavones in water, and in the case of *P. coccineus*, also on a solid MS medium.

The presence of flavones was demonstrated only in the plant mass (not in the medium), which proves the effective uptake of flavonoid compounds and biotransformation in the biocatalyst cells.

The addition of flavonoid compounds to aqueous plant cultures prolongs their stability, probably due to flavonoid compounds’ antioxidant activity.

Further research is necessary to reveal the full biocatalytic potential of the described callus cultures and to determine their potential applications.

## Figures and Tables

**Table 1:** Percentage of substrates and biotransformation products in *P. coccineus* water cultures.

	Substrate	Product
**Days**	Flavanone (**1**)	Flavone (**1a**)
7	99.4%	0.6%
14	98.9%	1.1%
	5-Methoxyflavanone (**2**)	5-Methoxyflavone (**2b**)
7	99.8%	0.2%
14	99.8%	0.2%
	6-Methoxyflavanone (**3**)	6-Methoxyflavone (**3a**)
7	98.9%	1.1%
14	97.7%	2.3%

**Table 2:** Percentage of substrates and biotransformation products in *G. max* water cultures.

	Substrate	Product
**Days**	Flavanone (**1**)	Flavone (**1a**)
7	98.7%	1.3%
14	97.4%	2.6%
	5-Methoxyflavanone (**2**)	5-Methoxyflavone (**2b**)
7	99.5%	0.5%
14	99.5%	0.5%
	6-Methoxyflavanone (**3**)	6-Methoxyflavone (**3a**)
7	95.3%	4.7%
14	92.5%	7.5%

**Table 3:** Percentage of substrates and biotransformation products in *P. coccineus* callus cultures on solid media.

Substrate	Product
Flavanone (**1**)	Flavone (**1a**)
99.7%	0.3%
5-Methoxyflavanone (**2**)	5-Methoxyflavone (**2b**)
98.7%	1.3%
6-Methoxyflavanone (**3**)	6-Methoxyflavone (**3a**)
98.5%	1.5%

## Supplementary Materials

The following are available online, Figure S1: The HPLC chromatogram of flavanone (**1**), Figure S2: The UV absorption maxima of flavanone (**1**), Figure S3: The HPLC chromatogram of flavone (**1a**), Figure S4: The UV absorption maxima of flavone (**1a**), Figure S5: The HPLC chromatogram of 5-methoxyflavanone (**2**), Figure S6: The UV absorption maxima of 5-methoxyflavanone (**2**), Figure S7: The HPLC chromatogram of 5-methoxyflavone (**2a**), Figure S8: The UV absorption maxima of 5-methoxyflavone (**2a**), Figure S9:The HPLC chromatogram of 6-methoxyflavanone (**3**), Figure S10: The UV absorption maxima of 6-methoxyflavanone (**3**), Figure S11: The HPLC chromatogram of 6-methoxyflavone (**3a**), Figure S12: The UV absorption maxima of 6-methoxyflavone (**3a**), Figure S13: The HPLC chromatogram—biotransformation of flavanone (**1**) in *Phaseolus coccineus* callus culture on solid medium, Figure S14: The UV absorption maxima of substrate (**1**) and product (**1a**) formed during biotransformation in *Phaseolus coccineus* callus culture on solid medium, Figure S15: The HPLC chromatogram—biotransformation of 5-methoxyflavanone (**2**) in *Phaseolus coccineus* callus culture on solid medium, Figure S16: The UV absorption maxima of substrate (**2**) and product (**2a**) formed during biotransformation in *Phaseolus coccineus* callus culture on solid medium, Figure S17: The HPLC chromatogram—biotransformation of 6-methoxyflavanone (**3**), in *Phaseolus coccineus* callus culture on solid medium, Figure S18: The UV absorption maxima of substrate (**3**) and product (**3a**) formed during biotransformation in *Phaseolus coccineus* callus culture on solid medium, Figure S19: The HPLC chromatogram—7-days biotransformation of flavanone (**1**) in *Phaseolus coccineus* callus water culture, Figure S20: The UV absorption maxima of substrate (**1**) and product (**1a**) formed during 7-days biotransformation in *Phaseolus coccineus* callus water culture, Figure S21: The HPLC chromatogram—14-days biotransformation of flavanone (**1**) in *Phaseolus coccineus* callus water culture, Figure S22: The UV absorption maxima of substrate (**1**) and product (**1a**) formed during 14-days biotransformation in *Phaseolus coccineus* callus water culture, Figure S23: The HPLC chromatogram—7-days biotransformation of 5-methoxyflavanone (**2**) in *Phaseolus coccineus* callus water culture, Figure S24: The UV absorption maxima of substrate (**2**) and product (**2a**) formed during 7-days biotransformation in *Phaseolus coccineus* callus water culture, Figure S25: The HPLC chromatogram—14-days biotransformation of 5-methoxyflavanone (**2**) in *Phaseolus coccineus* callus water culture, Figure S26: The UV absorption maxima of substrate (**2**) and product (**2a**) formed during 14-days biotransformation in *Phaseolus coccineus* callus water culture, Figure S27: The HPLC chromatogram—7-days biotransformation of 6-methoxyflavanone (**3**) in *Phaseolus coccineus* callus water culture, Figure S28: The UV absorption maxima of substrate (**3**) and product (**3a**) formed during 7-days biotransformation in *Phaseolus coccineus* callus water culture, Figure S29: The HPLC chromatogram—14-days biotransformation of 6-methoxyflavanone (**3**) in *Phaseolus coccineus* callus water culture, Figure S30: The UV absorption maxima of substrate (**3**) and product (**3a**) formed during 14-days biotransformation in *Phaseolus coccineus* callus water culture, Figure S31: The HPLC chromatogram—7-days biotransformation of flavanone (**1**) in *Glycine max* callus water culture, Figure S32: The UV absorption maxima of substrate (**1**) and product (**1a**) formed during 7-days biotransformation in *Glycine max* callus water culture, Figure S33: The HPLC chromatogram—14-days biotransformation of flavanone (**1**) in *Glycine max* callus water culture, Figure S34: The UV absorption maxima of substrate (**1**) and product (**1a**) formed during 14-days biotransformation in *Glycine max* callus water culture, Figure S35: The HPLC chromatogram—7-days biotransformation of 5-methoxyflavanone (**2**) in *Glycine max* callus water culture, Figure S36: The UV absorption maxima of substrate (**2**) and product (**2a**) formed during 7-days biotransformation in *Glycine max* callus water culture, Figure S37: The HPLC chromatogram—14-days biotransformation of 5-methoxyflavanone (**2**) in *Glycine max* callus water culture, Figure S38: The UV absorption maxima of substrate (**2**) and product (**2a**) formed during 14-days biotransformation in *Glycine max* callus water culture, Figure S39: The HPLC chromatogram—7-days biotransformation of 6-methoxyflavanone (**3**) in *Glycine max* callus water culture, Figure S40: The UV absorption maxima of substrate (**3**) and product (**3a**) formed during 7-days biotransformation in *Glycine max* callus water culture, Figure S41: The HPLC chromatogram—14-days biotransformation of 6-methoxyflavanone (**3**) in *Glycine max* callus water culture, Figure S42: The UV absorption maxima of substrate (**3**) and product (**3a**) formed during 14-days biotransformation in *Glycine max* callus water culture, Figure S43: The HPLC chromatogram—metabolites formed after 14 days in *Phaseolus coccineus* callus water culture (without substrate), Figure S44: The UV absorption maxima of *Phaseolus coccineus* metabolites, Figure S45: The HPLC chromatogram—metabolites formed after 14 days in *Glycine max* callus water culture (without substrate), Figure S46: The UV absorption maxima of *Glycine max* metabolites.
