# Supplementary material for: The Callus of Phaseolus coccineus and Glycine max Biotransform Flavanones into the Corresponding Flavones
Source: Molecules. 2020 Dec 7;25(23):5767. doi: 10.3390/molecules25235767 (PMC7730475; doi:10.3390/molecules25235767)
Supplement: Supplementary file 1 [file molecules-25-05767-s001.pdf]

molecules

Supplementary data

The callus of *Phaseolus coccineus* and *Glycine max* converts flavanones into the corresponding flavones

Monika Dymarska *, Tomasz Janeczko and Edyta Kostrzewa - Susłow

Department of Chemistry, Wrocław University of Environmental and Life Sciences, Norwida 25, Wrocław 50-375, Poland; E-Mails : ekostrzew@gmail.com (E. K.-S.), janeczko13@interia.pl (T. J.)

* Correspondence: monika.dymarska@gmail.com

Figure S1 The HPLC chromatogram of flavanone (1)

Figure S2 The UV absorption maxima of flavanone (1)

Figure S3 The HPLC chromatogram of flavone (1a)

Figure S4 The UV absorption maxima of flavone (1a)

Figure S5 The HPLC chromatogram of 5-methoxyflavanone (2)

Figure S6 The UV absorption maxima of 5-methoxyflavanone (2)

Figure S7 The HPLC chromatogram of 5-methoxyflavone (2a)

Figure S8 The UV absorption maxima of 5-methoxyflavone (2a)

Figure S9 The HPLC chromatogram of 6-methoxyflavanone (3)

Figure S10 The UV absorption maxima of 6-methoxyflavanone (3)

Figure S11 The HPLC chromatogram of 6-methoxyflavone (3a)

Figure S12 The UV absorption maxima of 6-methoxyflavone (3a)

Figure S13 The HPLC chromatogram – biotransformation of flavanone (1) in *Phaseolus coccineus* callus culture on solid medium

Figure S14 The UV absorption maxima of substrate (1) and product (1a) formed during biotransformation in *Phaseolus coccineus* callus culture on solid medium

Figure S15 The HPLC chromatogram – biotransformation of 5-methoxyflavanone (2) in *Phaseolus coccineus* callus culture on solid medium

Figure S16 The UV absorption maxima of substrate (2) and product (2a) formed during biotransformation in *Phaseolus coccineus* callus culture on solid medium

Figure S17 The HPLC chromatogram – biotransformation of 6-methoxyflavanone (3) in *Phaseolus coccineus* callus culture on solid medium

Figure S18 The UV absorption maxima of substrate (3) and product (3a) formed during biotransformation in *Phaseolus coccineus* callus culture on solid medium

Figure S19 The HPLC chromatogram – 7-days biotransformation of flavanone (1) in *Phaseolus coccineus* callus water culture

Figure S20 The UV absorption maxima of substrate (1) and product (1a) formed during 7-days biotransformation in *Phaseolus coccineus* callus water culture

Figure S21 The HPLC chromatogram – 14-days biotransformation of flavanone (1) in *Phaseolus coccineus* callus water culture

Figure S22 The UV absorption maxima of substrate (1) and product (1a) formed during 14-days biotransformation in *Phaseolus coccineus* callus water culture

Figure S23 The HPLC chromatogram – 7-days biotransformation of 5-methoxyflavanone (2) in *Phaseolus coccineus* callus water culture
Figure S24 The UV absorption maxima of substrate (2) and product (2a) formed during 7-days biotransformation in *Phaseolus coccineus* callus water culture
Figure S25 The HPLC chromatogram – 14-days biotransformation of 5-methoxyflavanone (2) in *Phaseolus coccineus* callus water culture
Figure S26 The UV absorption maxima of substrate (2) and product (2a) formed during 14-days biotransformation in *Phaseolus coccineus* callus water culture
Figure S27 The HPLC chromatogram – 7-days biotransformation of 6-methoxyflavanone (3) in *Phaseolus coccineus* callus water culture
Figure S28 The UV absorption maxima of substrate (3) and product (3a) formed during 7-days biotransformation in *Phaseolus coccineus* callus water culture
Figure S29 The HPLC chromatogram – 14-days biotransformation of 6-methoxyflavanone (3) in *Phaseolus coccineus* callus water culture
Figure S30 The UV absorption maxima of substrate (3) and product (3a) formed during 14-days biotransformation in *Phaseolus coccineus* callus water culture
Figure S31 The HPLC chromatogram – 7-days biotransformation of flavanone (1) in *Glycine max* callus water culture
Figure S32 The UV absorption maxima of substrate (1) and product (1a) formed during 7-days biotransformation in *Glycine max* callus water culture
Figure S33 The HPLC chromatogram – 14-days biotransformation of flavanone (1) in *Glycine max* callus water culture
Figure S34 The UV absorption maxima of substrate (1) and product (1a) formed during 14-days biotransformation in *Glycine max* callus water culture
Figure S35 The HPLC chromatogram – 7-days biotransformation of 5-methoxyflavanone (2) in *Glycine max* callus water culture
Figure S36 The UV absorption maxima of substrate (2) and product (2a) formed during 7-days biotransformation in *Glycine max* callus water culture
Figure S37 The HPLC chromatogram – 14-days biotransformation of 5-methoxyflavanone (2) in *Glycine max* callus water culture
Figure S38 The UV absorption maxima of substrate (2) and product (2a) formed during 14-days biotransformation in *Glycine max* callus water culture
Figure S39 The HPLC chromatogram – 7-days biotransformation of 6-methoxyflavanone (3) in *Glycine max* callus water culture
Figure S40 The UV absorption maxima of substrate (3) and product (3a) formed during 7-days biotransformation in *Glycine max* callus water culture
Figure S41 The HPLC chromatogram – 14-days biotransformation of 6-methoxyflavanone (3) in *Glycine max* callus water culture
Figure S42 The UV absorption maxima of substrate (3) and product (3a) formed during 14-days biotransformation in *Glycine max* callus water culture
Figure S43 The HPLC chromatogram – metabolites formed after 14 days in *Phaseolus coccineus* callus water culture (without substrate)
Figure S44 The UV absorption maxima of *Phaseolus coccineus* metabolites

molecules

MDPI

Figure S45 The HPLC chromatogram – metabolites formed after 14 days in *Glycine max* callus water culture (without substrate)

Figure S46 The UV absorption maxima of *Glycine max* metabolites

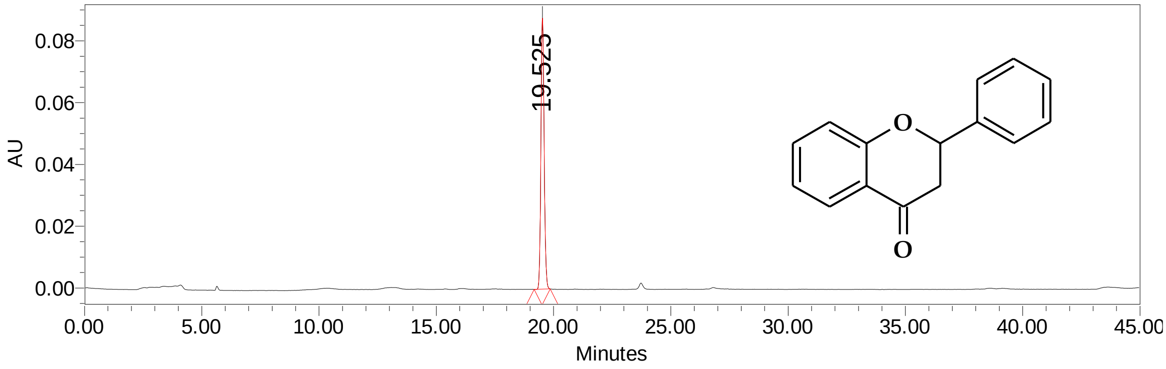

Figure S1 The HPLC chromatogram of flavanone (1)

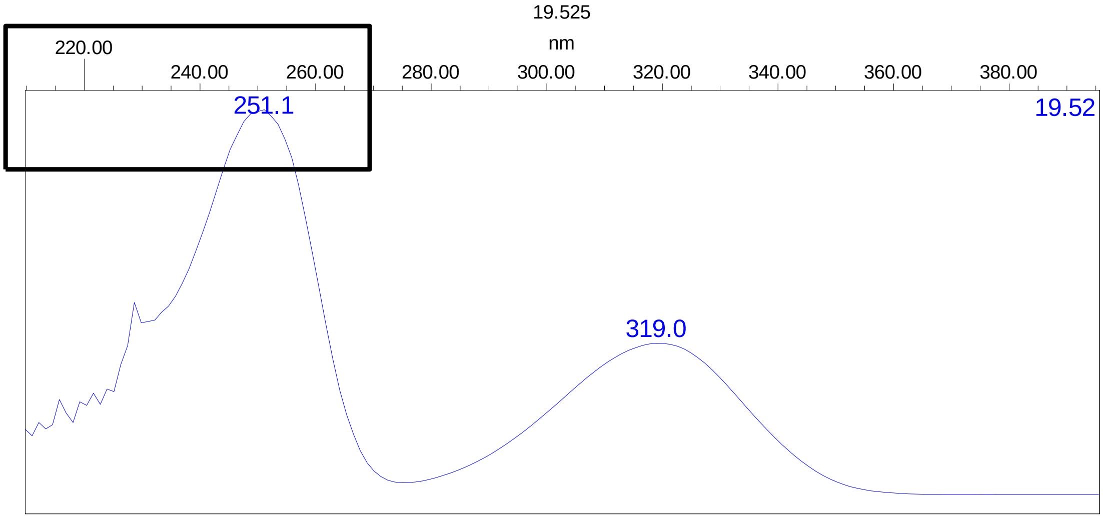

Figure S2 The UV absorption maxima of flavanone (1)

molecules

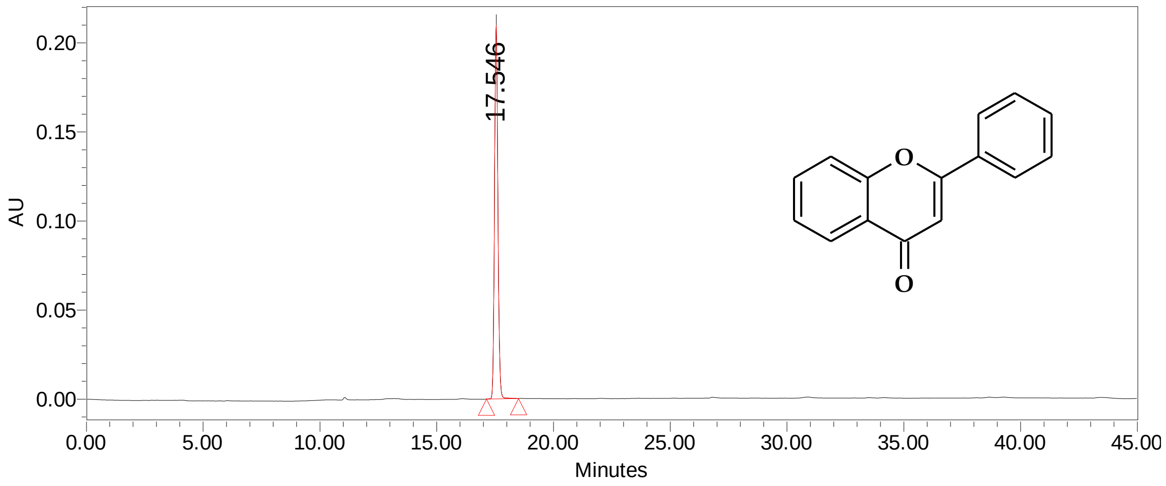

Figure S3 The HPLC chromatogram of flavone (1a)

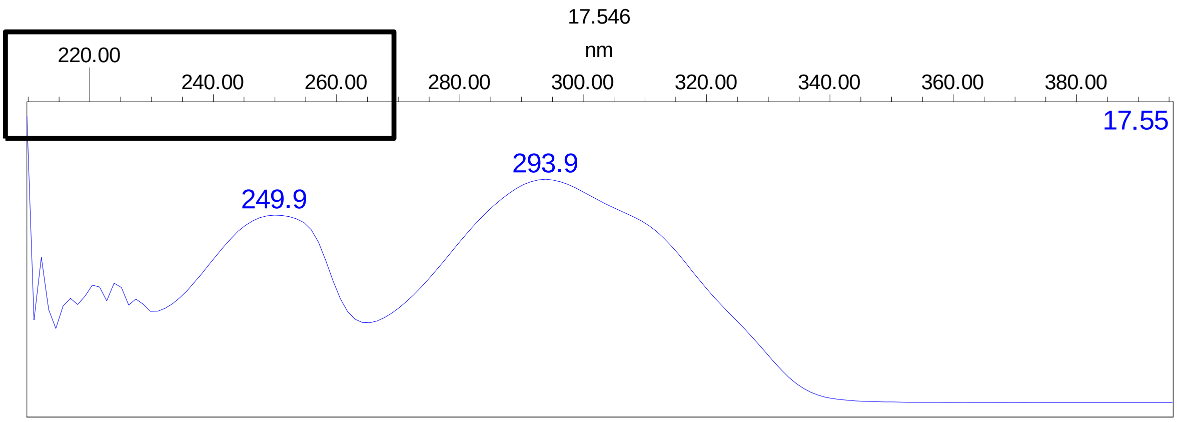

Figure S4 The UV absorption maxima of flavone (1a)

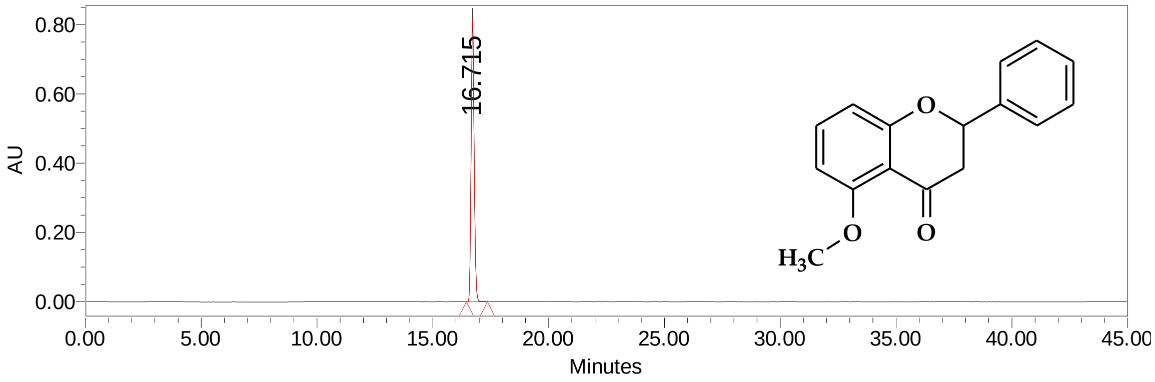

Figure S5 The HPLC chromatogram of 5-methoxyflavanone (2)

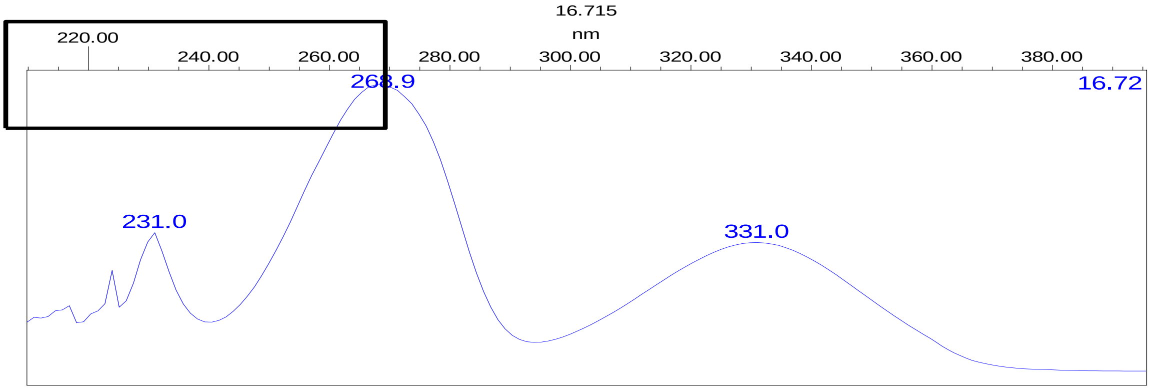

Figure S6 The UV absorption maxima of 5-methoxyflavanone (2)

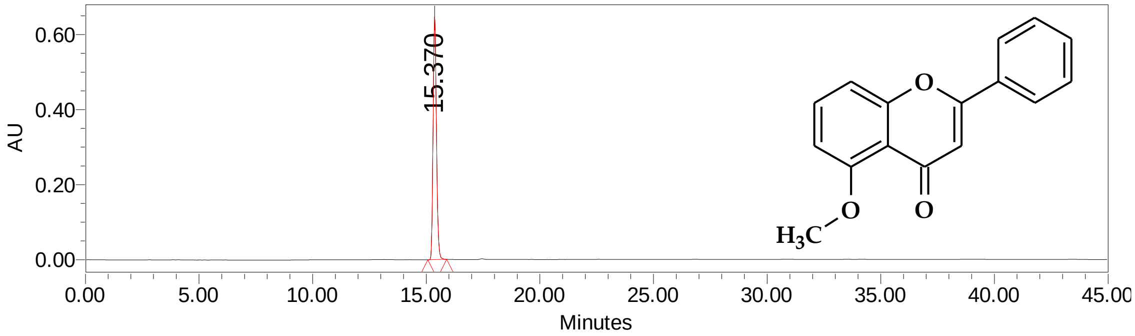

Figure S7 The HPLC chromatogram of 5-methoxyflavone (2a)

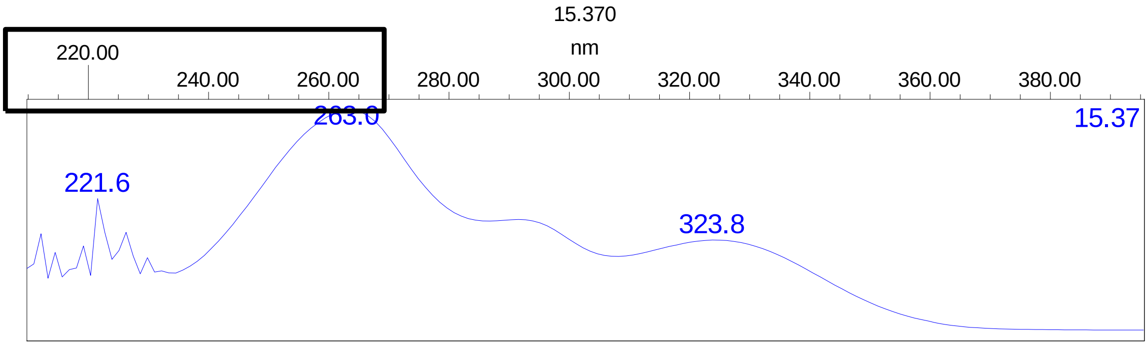

Figure S8 The UV absorption maxima of 5-methoxyflavone (2a)

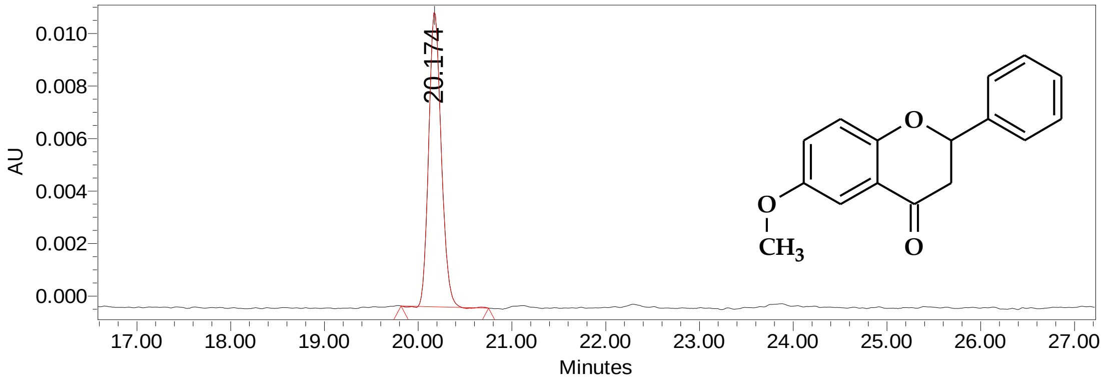

Figure S9 The HPLC chromatogram of 6-methoxyflavanone (3)

molecules

MDPI

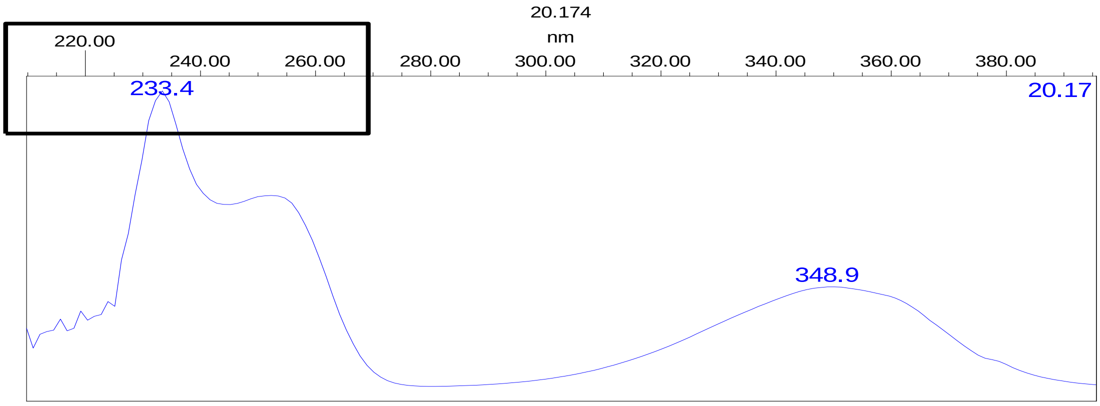

Figure S10 The UV absorption maxima of 6-methoxyflavanone (3)

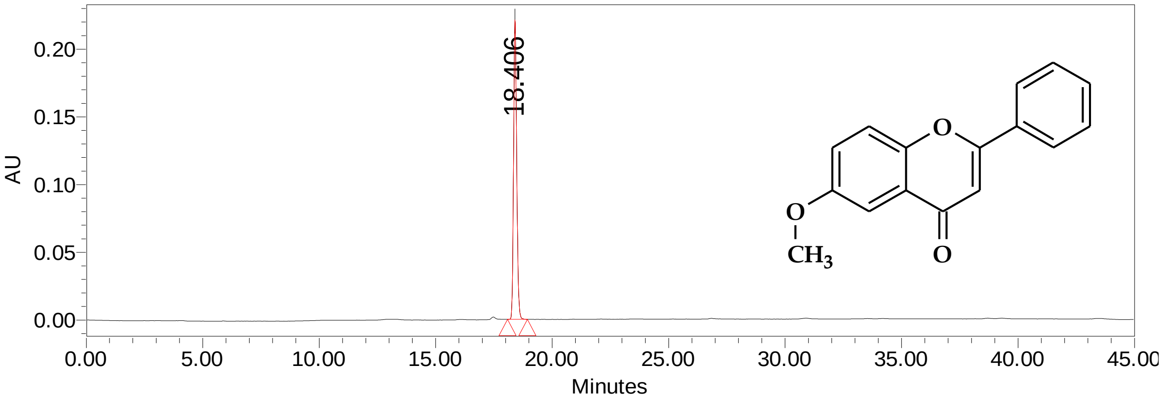

Figure S11 The HPLC chromatogram of 6-methoxyflavone (3a)

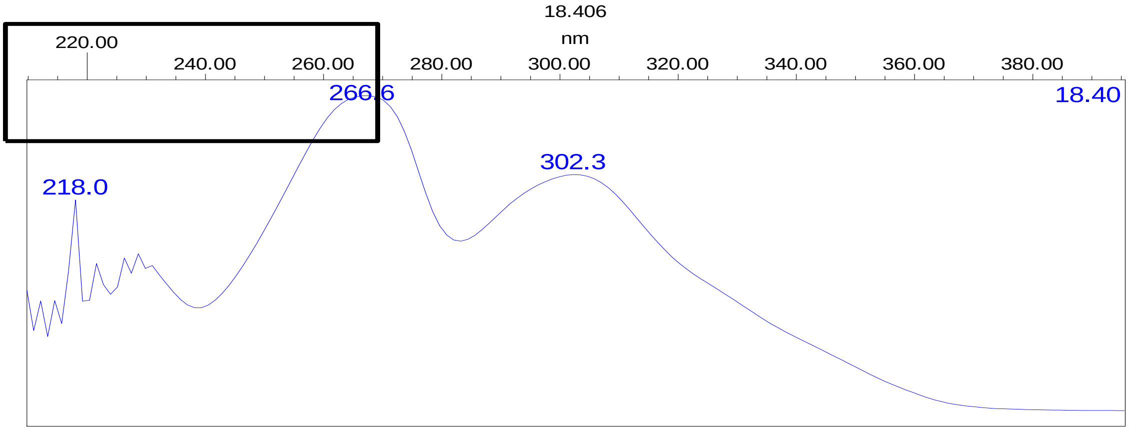

Figure S12 The UV absorption maxima of 6-methoxyflavone (3a)

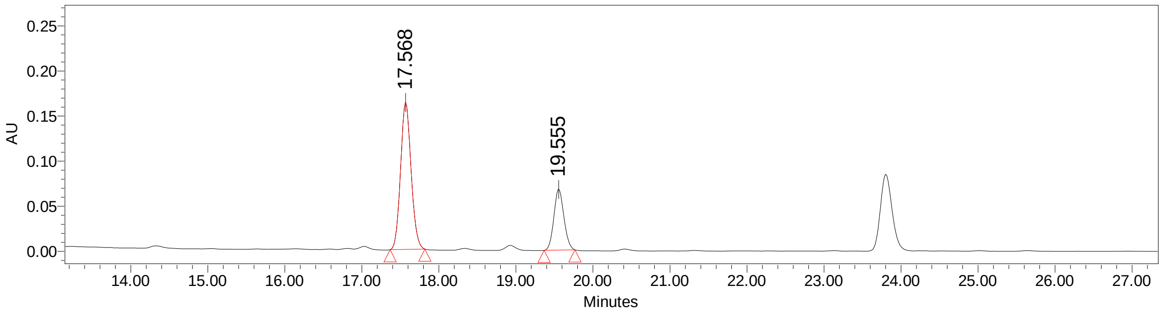

Figure S13 The HPLC chromatogram – biotransformation of flavanone (1) in *Phaseolus coccineus* callus culture on solid medium; t_R = 19,6 (flavanone-substrate (1), t_R = 17,6 (flavone-product (1a).

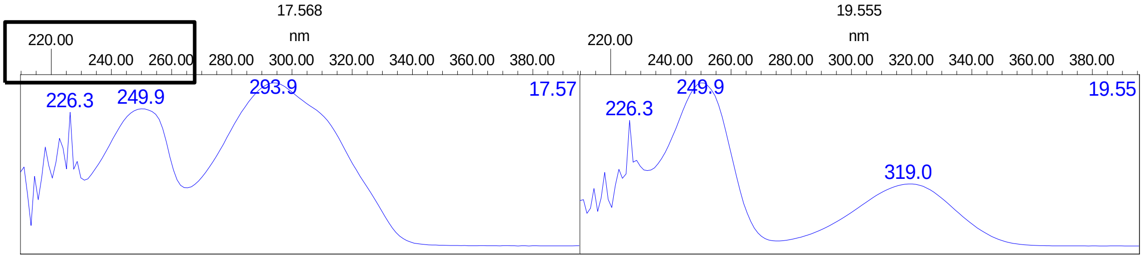

Figure S14 The UV absorption maxima of flavanone-substrate (1) and flavone-product (1a) formed during biotransformation in *Phaseolus coccineus* callus culture on solid medium

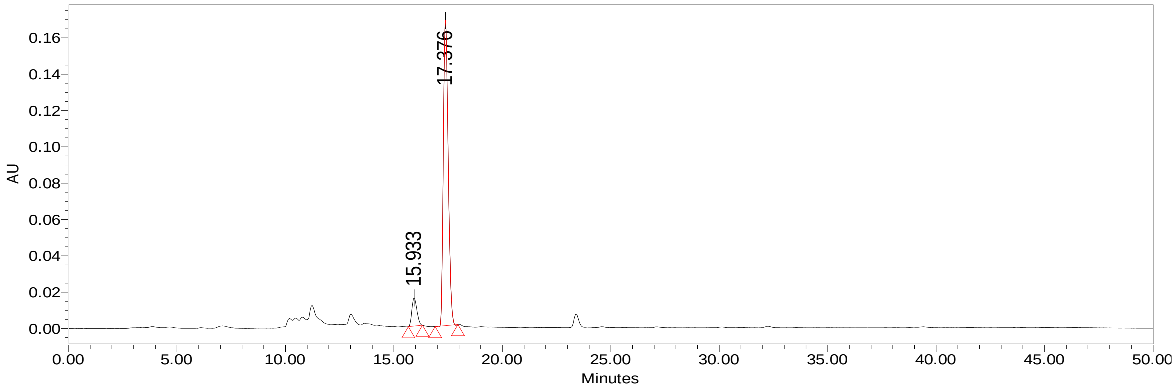

Figure S15 The HPLC chromatogram – biotransformation of 5-methoxyflavanone (2) in *Phaseolus coccineus* callus culture on solid medium; t_R = 17,4 (5-methoxyflavanone-substrate (2), t_R = 15,9 (5-methoxyflavone-product (2a).

molecules MDPI

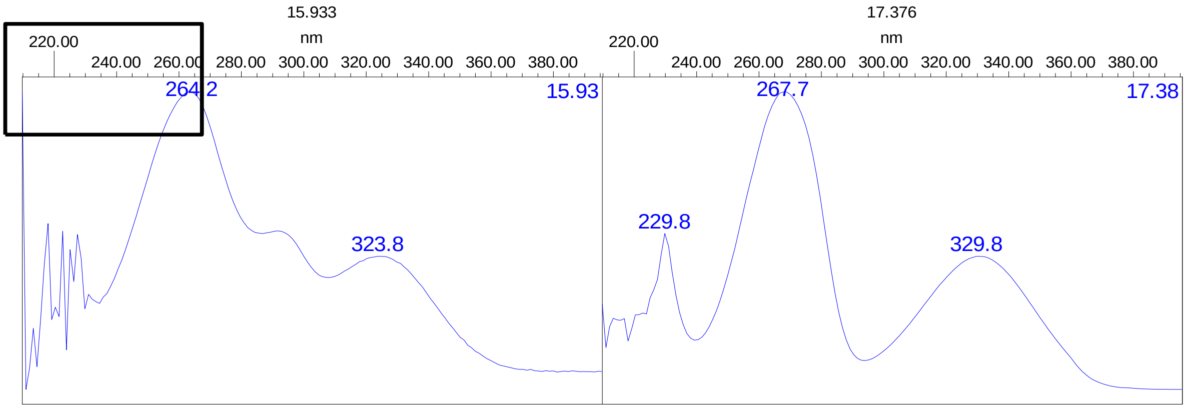

Figure S16 The UV absorption maxima of 5-methoxyflavanone-substrate (2) and 5-methoxyflavone-product (2a) formed during biotransformation in *Phaseolus coccineus* callus culture on solid medium

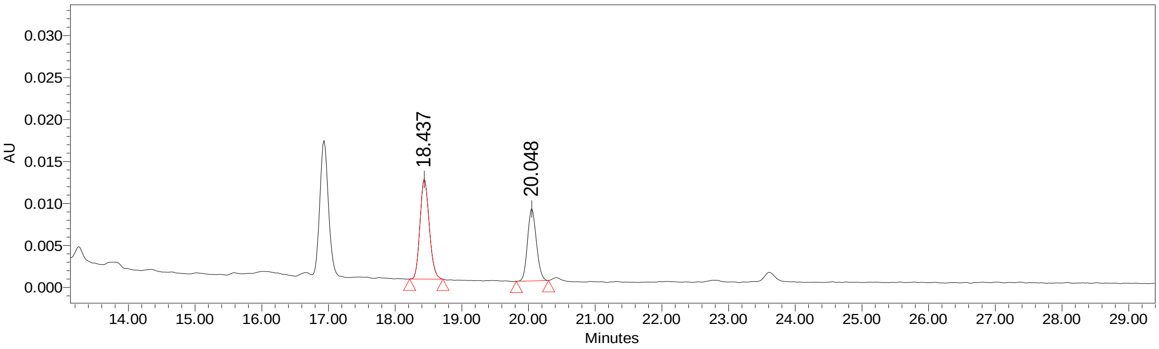

Figure S17 The HPLC chromatogram – biotransformation of 6-methoxyflavanone (3) in *Phaseolus coccineus* callus culture on solid medium; t_R = 20,0 (6-methoxyflavanone-substrate (3), t_R = 18,4 (6-methoxyflavone-product (3a).

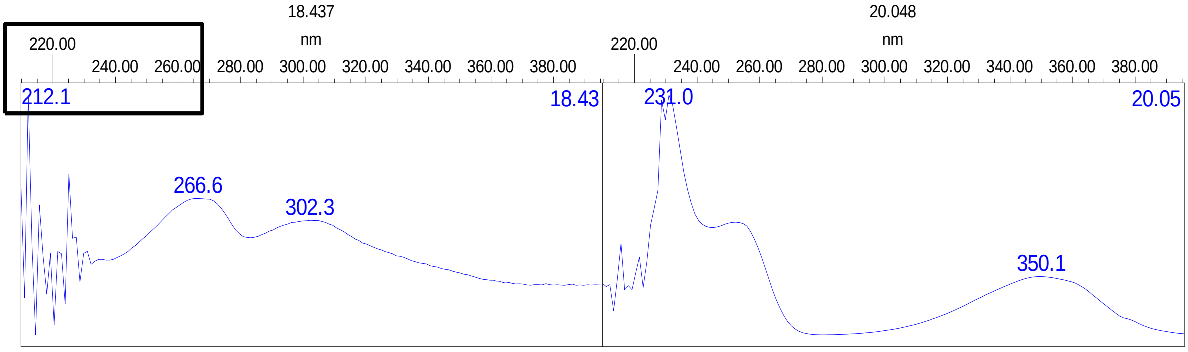

Figure S18 The UV absorption maxima of 6-methoxyflavanone-substrate (3) and 6-methoxyflavone-product (3a) formed during biotransformation in *Phaseolus coccineus* callus culture on solid medium

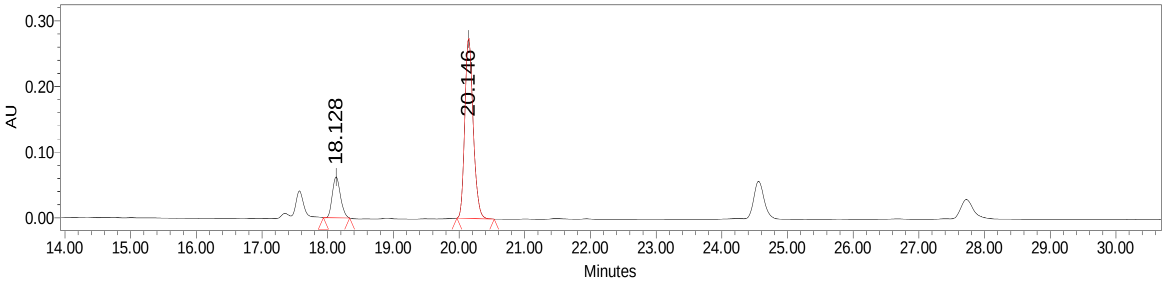

Figure S19 The HPLC chromatogram – 7-days biotransformation of flavanone (1) in *Phaseolus coccineus* callus water culture.

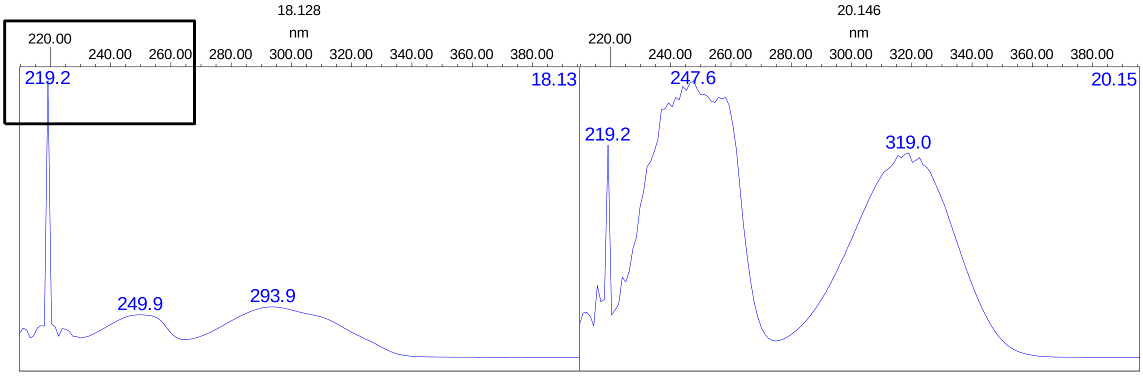

Figure S20 The UV absorption maxima of flavanone-substrate (1) and flavone-product (1a) formed during biotransformation in *Phaseolus coccineus* callus water culture

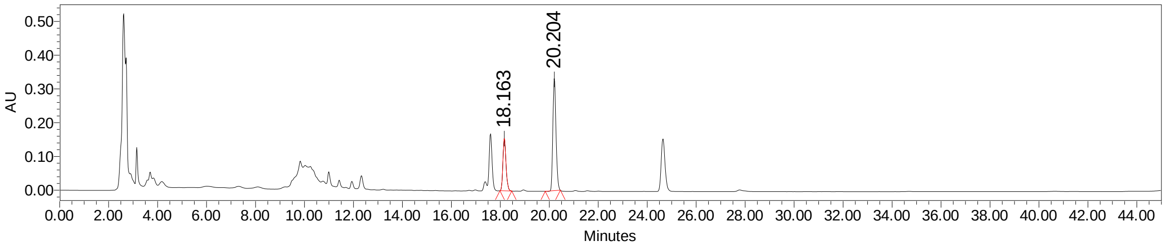

Figure S21 The HPLC chromatogram – 14-days biotransformation of flavanone (1) in *Phaseolus coccineus* callus water culture; t_R = 20,2 (flavanone-substrate (1), t_R = 18,2 (flavone-product (1a).

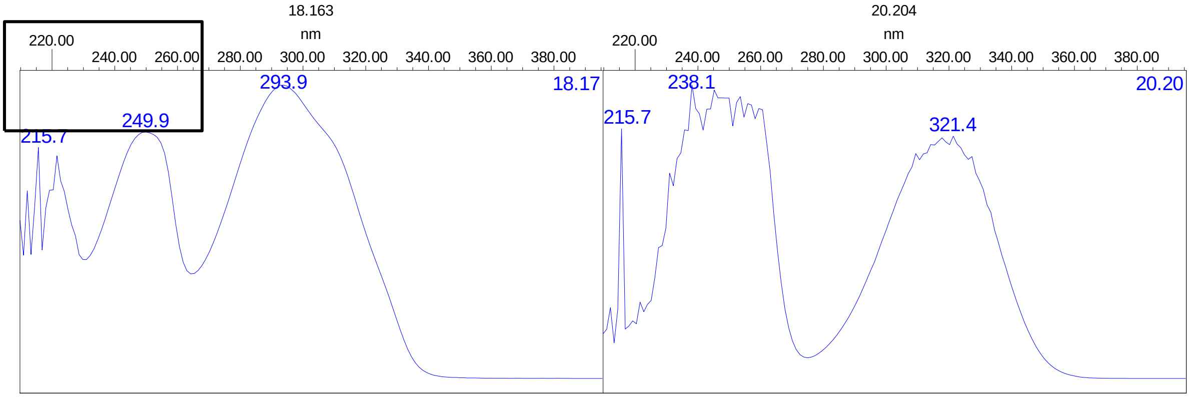

Figure S22 The UV absorption maxima of flavanone-substrate (1) and flavone-product (1a) formed during 14-days biotransformation in *Phaseolus coccineus* callus water culture

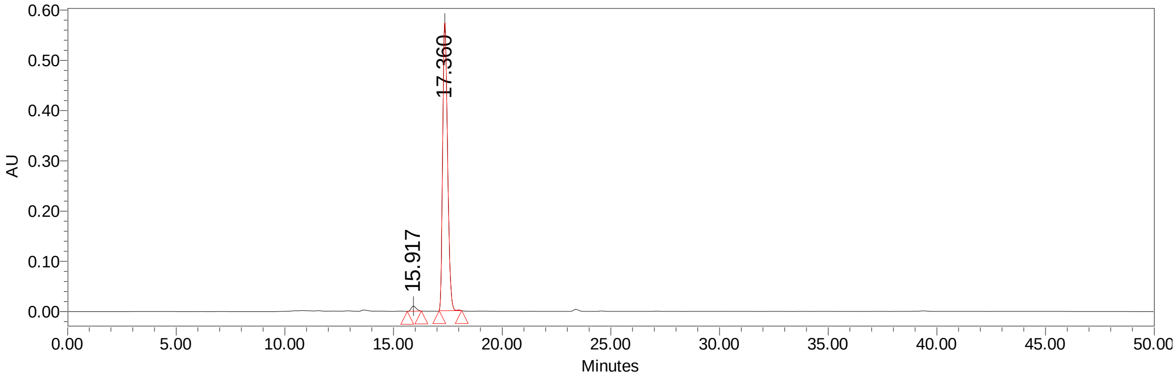

Figure S23 The HPLC chromatogram – 7-days biotransformation of 5-methoxyflavanone (2) in *Phaseolus coccineus* callus water culture; t_R = 17,4 (5-methoxyflavanone-substrate (2), t_R = 15,9 (5-methoxyflavone-product (2a).

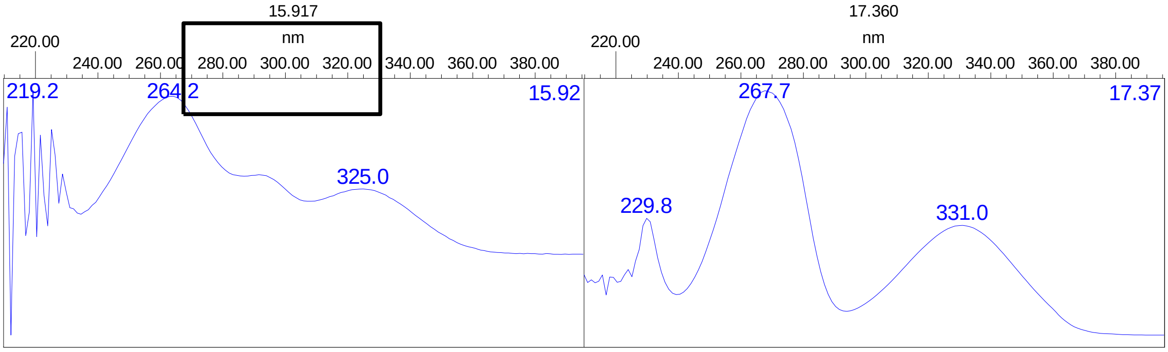

Figure S24 The UV absorption maxima of 5-methoxyflavanone-substrate (2) and 5-methoxyflavone-product (2a) formed during 7-days biotransformation in *Phaseolus coccineus* callus water culture

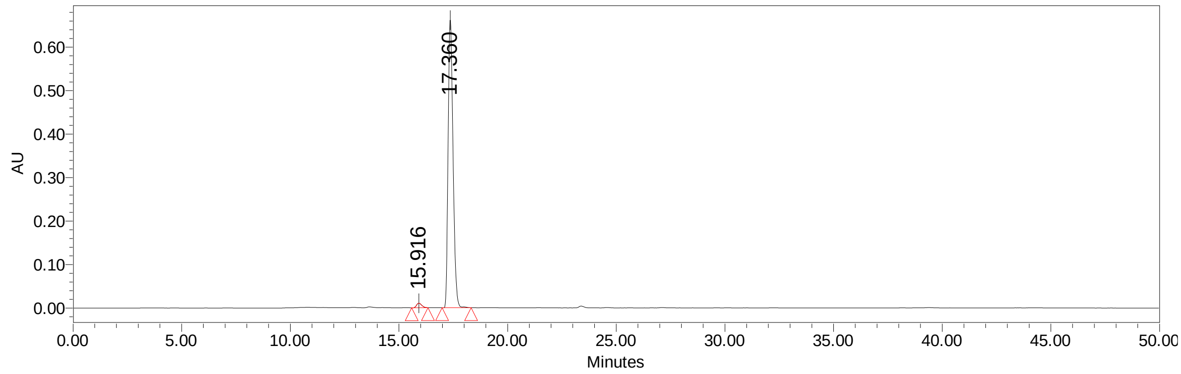

Figure S25 The HPLC chromatogram – 14-days biotransformation of 5-methoxyflavanone (2) in *Phaseolus coccineus* callus water culture; t_R = 17,4 (5-methoxyflavanone-substrate (2), t_R = 15,9 (5-methoxyflavone-product (2a).

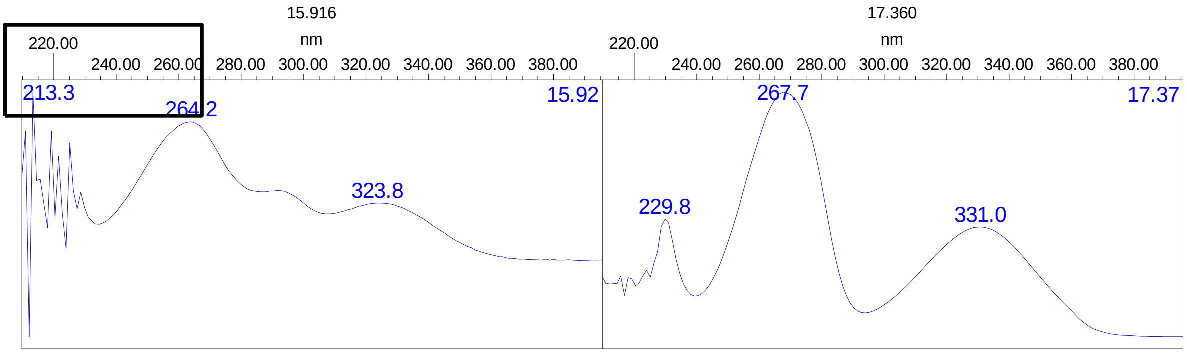

Figure S26 The UV absorption maxima of 5-methoxyflavanone-substrate (2) and 5-methoxyflavone-product (2a) formed during 14-days biotransformation in *Phaseolus coccineus* callus water culture

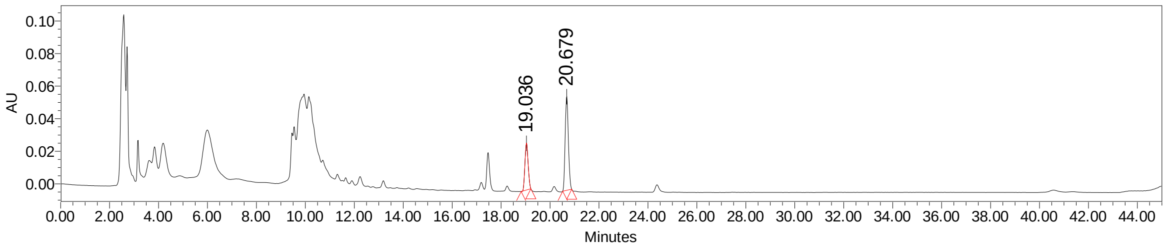

Figure S27 The HPLC chromatogram – 7-days biotransformation of 6-methoxyflavanone (3) in *Phaseolus coccineus* callus water culture; t_R = 20,7 (6-methoxyflavanone-substrate (3), t_R = 19,0 (6-methoxyflavone-product (3a).

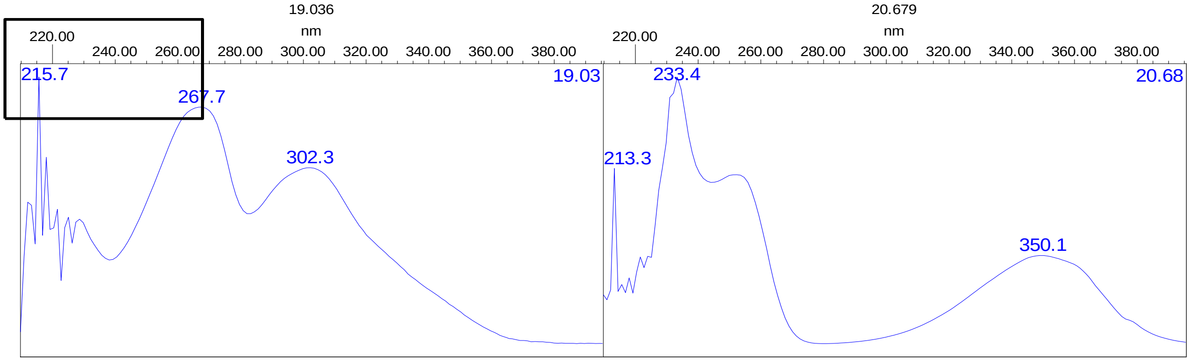

Figure S28 The UV absorption maxima of 6-methoxyflavanone-substrate (3) and 6-methoxyflavone-product (3a) formed during 7-days biotransformation in *Phaseolus coccineus* callus water culture

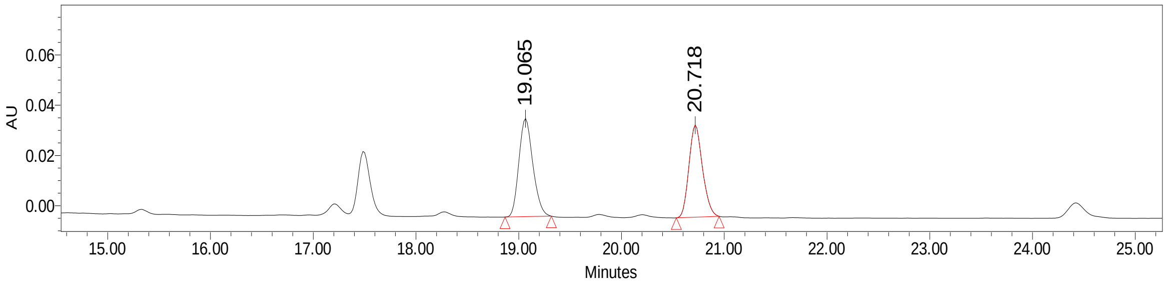

Figure S29 The HPLC chromatogram – 14-days biotransformation of 6-methoxyflavanone (3) in *Phaseolus coccineus* callus water culture; t_R = 20,7 (6-methoxyflavanone-substrate (3), t_R = 19,1 (6-methoxyflavone-product (3a).

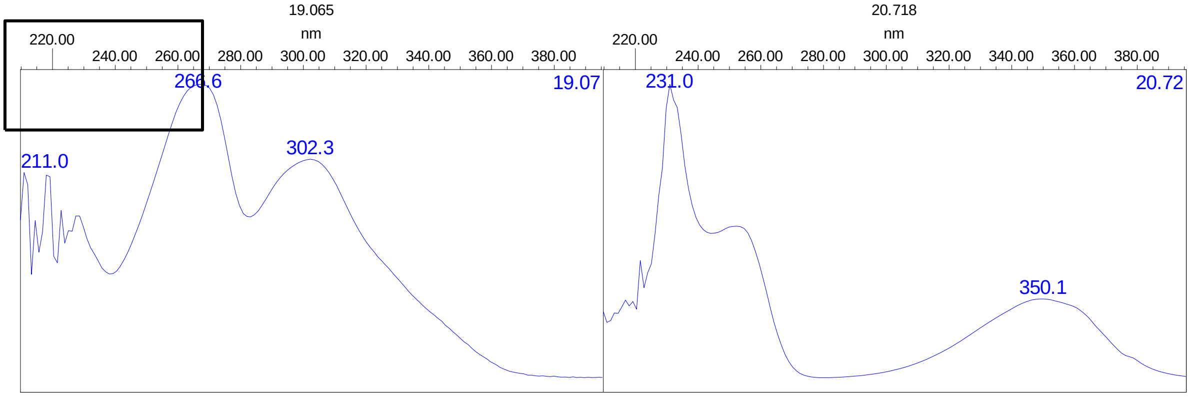

Figure S30 The UV absorption maxima of 6-methoxyflavanone-substrate (3) and 6-methoxyflavone-product (3a) formed during 14-days biotransformation in *Phaseolus coccineus* callus water culture

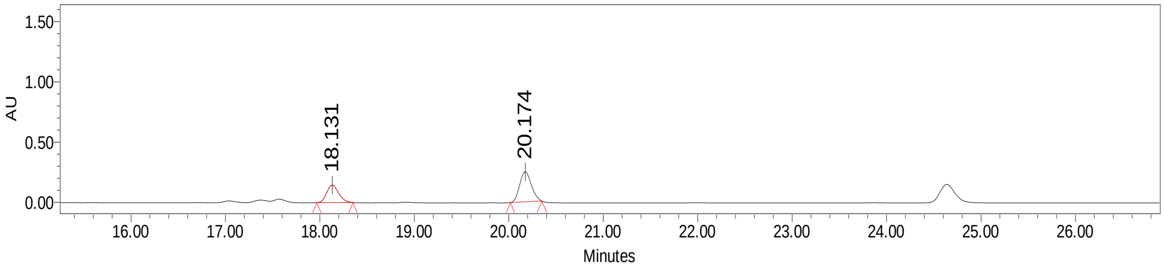

Figure S31 The HPLC chromatogram – 7-days biotransformation of flavanone (1) in *Glycine max* callus water culture; t_R = 20,2 (flavanone-substrate (1), t_R = 18,1 (flavone-product (1a).

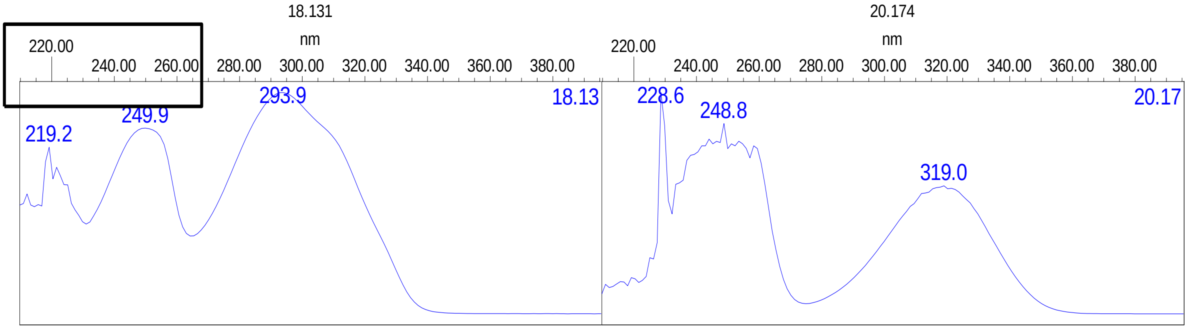

Figure S32 The UV absorption maxima of flavanone-substrate (1) and flavone-product (1a) formed during 7-days biotransformation in *Glycine max* callus water culture

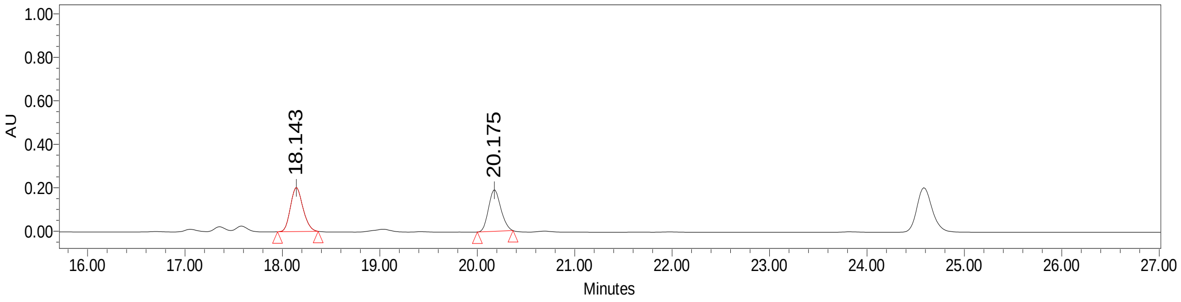

Figure S33 The HPLC chromatogram – 14-days biotransformation of flavanone (1) in *Glycine max* callus water culture; t_R = 20,2 (flavanone-substrate (1), t_R = 18,1 (flavone-product (1a).

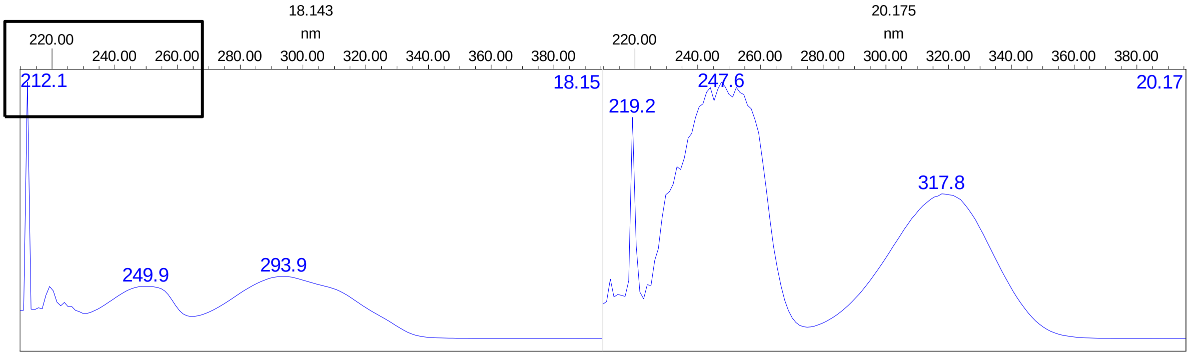

Figure S34 The UV absorption maxima of flavanone-substrate (1) and flavone-product (1a) formed during 14-days biotransformation in *Glycine max* callus water culture

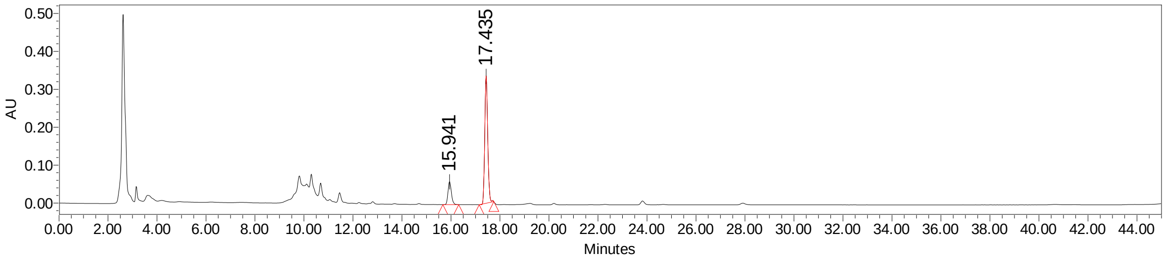

Figure S35 The HPLC chromatogram – 7-days biotransformation of 5-methoxyflavanone (2) in *Glycine max* callus water culture; t_R = 17,4 (5-methoxyflavanone-substrate (2), t_R = 15,9 (5-methoxyflavone-product (2a).

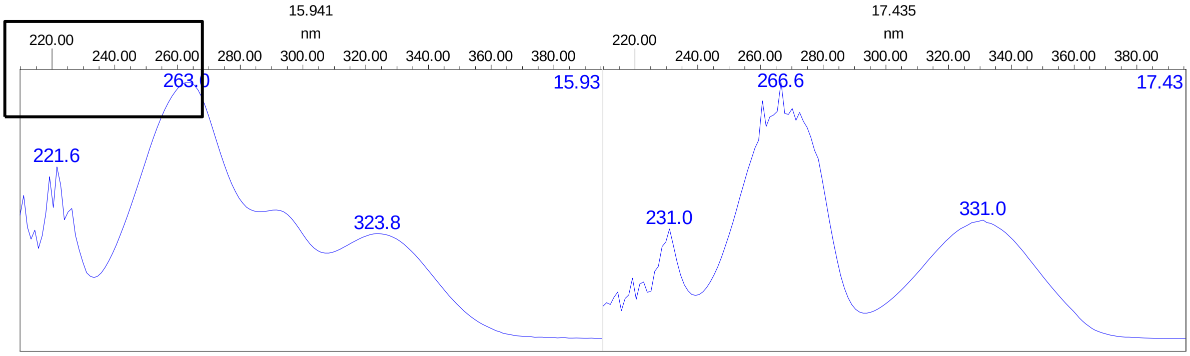

Figure S36 The UV absorption maxima of 5-methoxyflavanone-substrate (2) and 5-methoxyflavone-product (2a) formed during 7-days biotransformation in *Glycine max* callus water culture

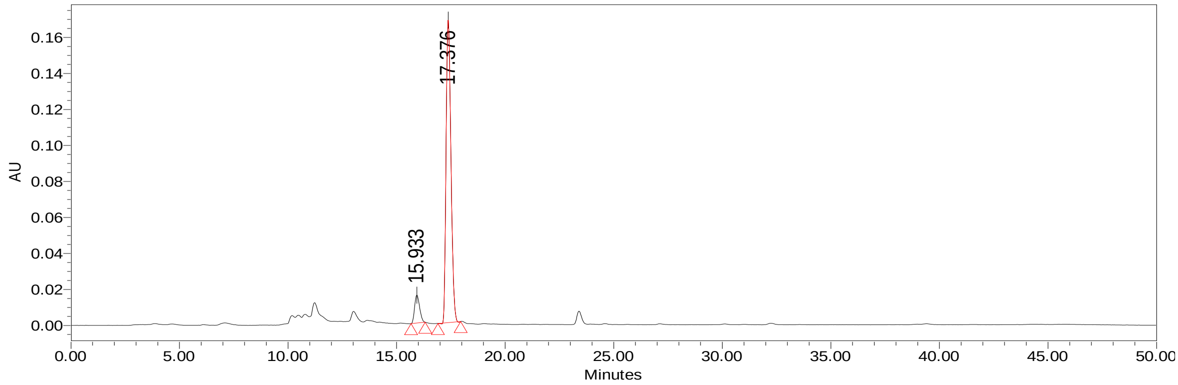

Figure S37 The HPLC chromatogram – 14-days biotransformation of 5-methoxyflavanone (2) in *Glycine max* callus water culture; t_R = 17,4 (5-methoxyflavanone-substrate (2), t_R = 15,9 (5-methoxyflavone-product (2a).

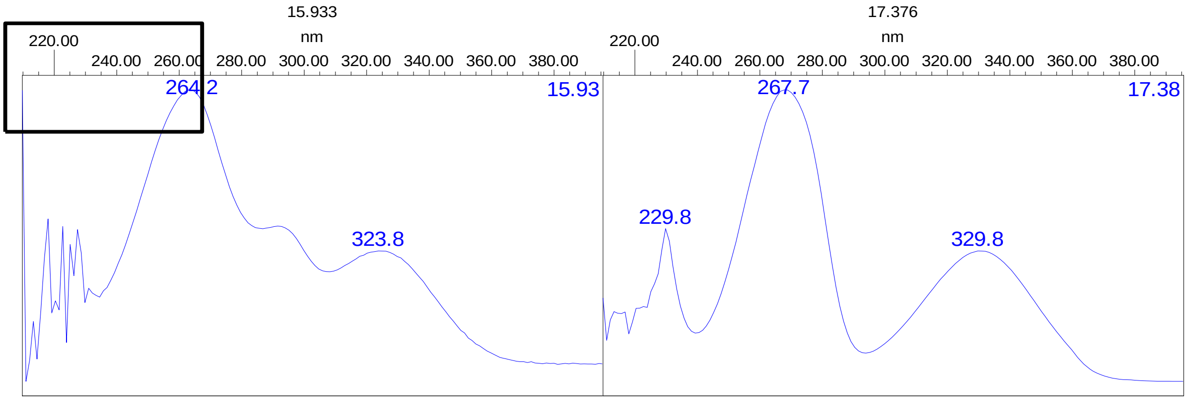

Figure S38 The UV absorption maxima of 5-methoxyflavanone-substrate (2) and 5-methoxyflavone-product (2a) formed during 14-days biotransformation in *Glycine max* callus water culture

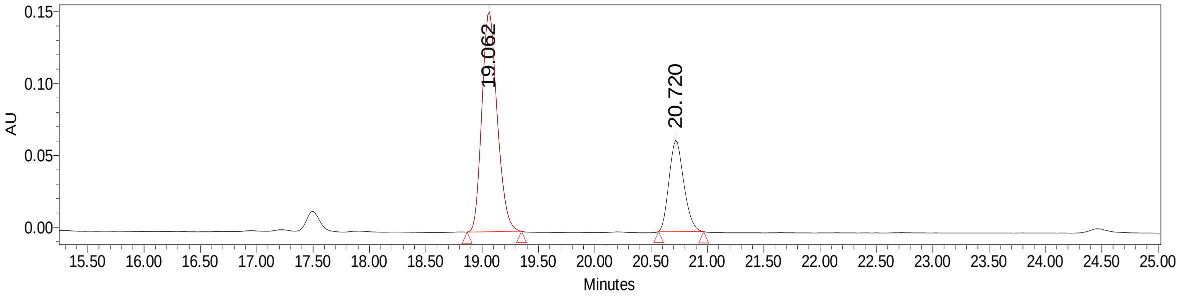

Figure S39 The HPLC chromatogram – 7-days biotransformation of 6-methoxyflavanone (3) in *Glycine max* callus water culture; t_R = 20,7 (6-methoxyflavanone-substrate (3), t_R = 19,1 (6-methoxyflavone-product (3a).

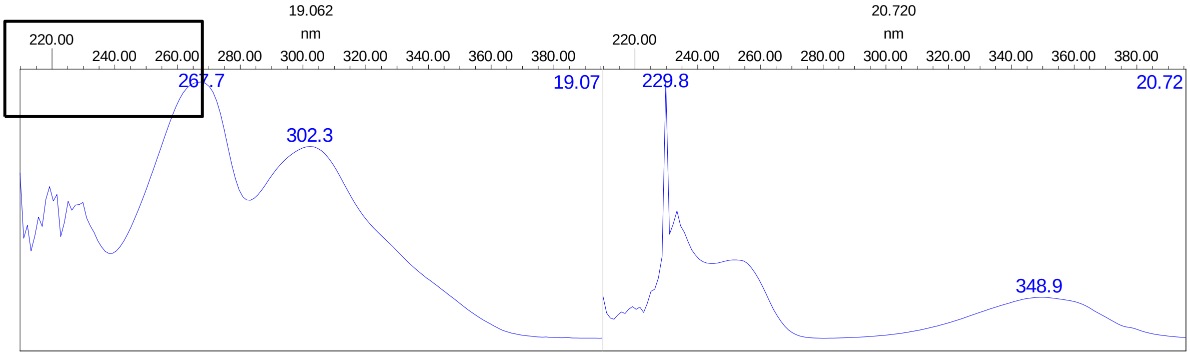

Figure S40 The UV absorption maxima of 6-methoxyflavanone-substrate (3) and 6-methoxyflavone-product (3a) formed during 7-days biotransformation in *Glycine max* callus water culture

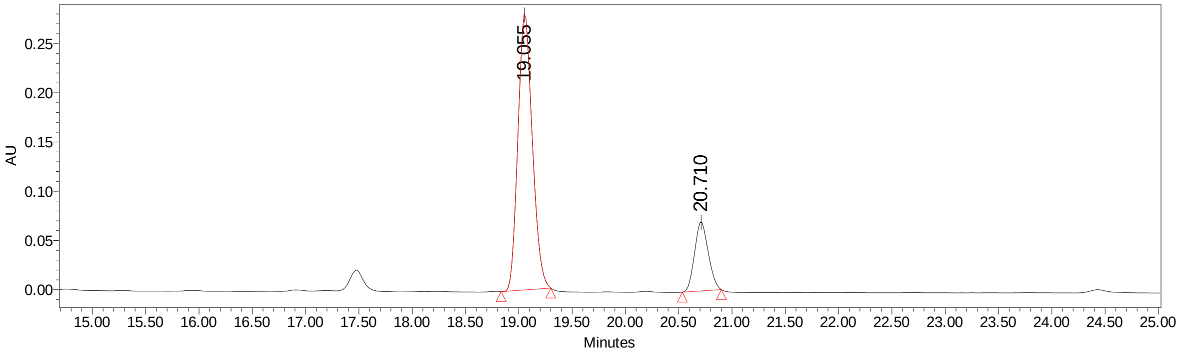

Figure S41 The HPLC chromatogram – 14-days biotransformation of 6-methoxyflavanone (3) in *Glycine max* callus water culture; t_R = 20,7 (6-methoxyflavanone-substrate (3), t_R = 19,1 (6-methoxyflavone-product (3a).

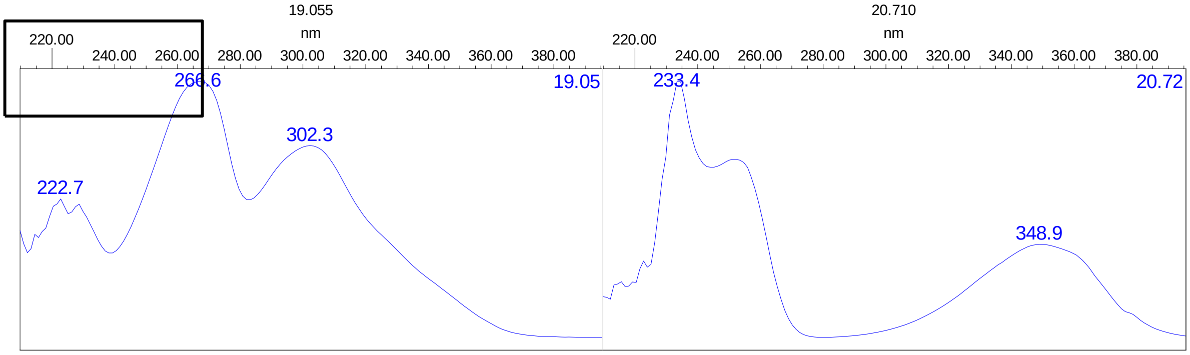

Figure S42 The UV absorption maxima of 6-methoxyflavanone-substrate (3) and 6-methoxyflavone-product (3a) formed during 14-days biotransformation in *Glycine max* callus water culture

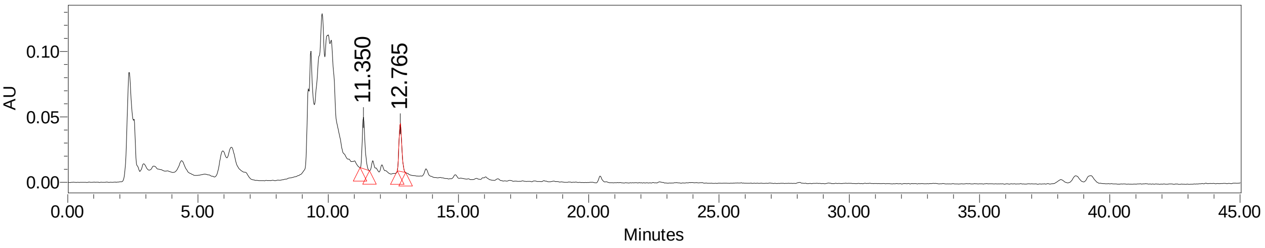

Figure S43 The HPLC chromatogram – metabolites formed after 14 days in *Phaseolus coccineus* callus water culture (without substrate)

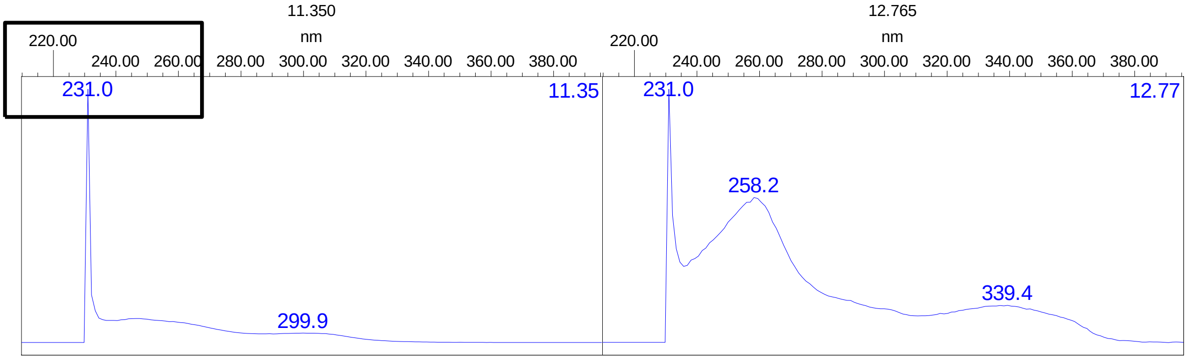

Figure S44 The UV absorption maxima of *Phaseolus coccineus* metabolites

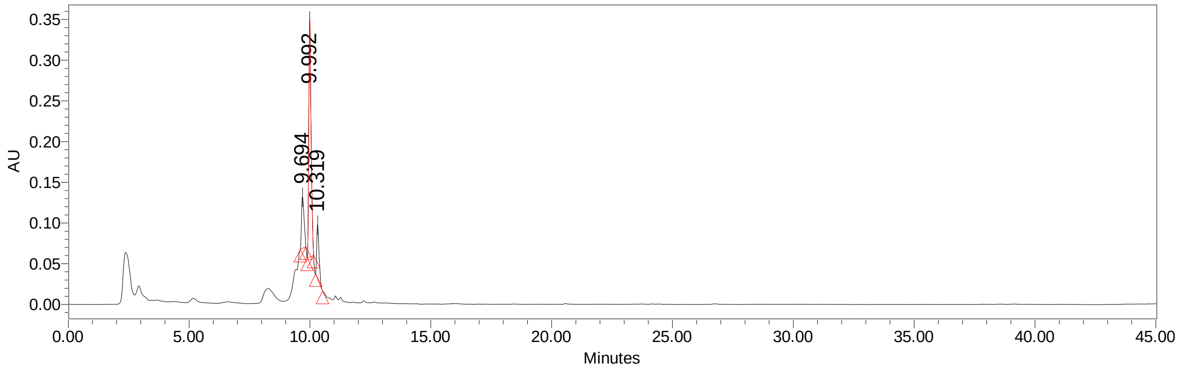

Figure S45 The HPLC chromatogram – metabolites formed after 14 days in *Glycine max* callus water culture (without substrate)

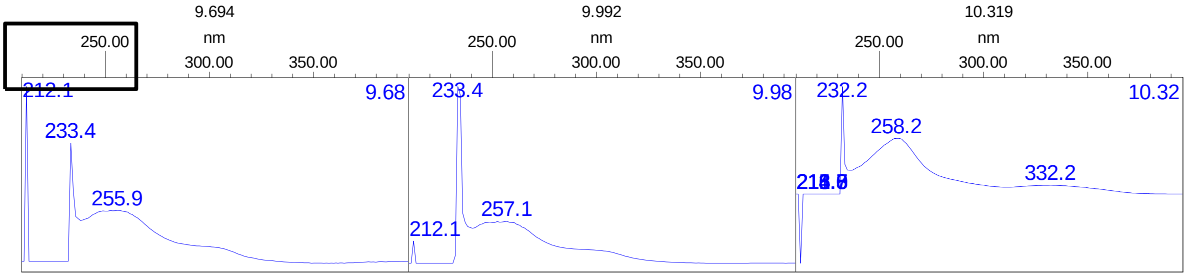

Figure S46 The UV absorption maxima of *Glycine max* metabolites
